# An Integrated *In Vitro* Imaging Platform for Characterizing Filarial Parasite Behavior within a Multicellular Microenvironment

**DOI:** 10.1371/journal.pntd.0003305

**Published:** 2014-11-20

**Authors:** Timothy Kassis, Henry M. Skelton, Iris M. Lu, Andrew R. Moorhead, J. Brandon Dixon

**Affiliations:** 1 Parker H. Petit Institute for Bioengineering & Bioscience, Georgia Institute of Technology, Atlanta, Georgia, United States of America; 2 School of Electrical and Computer Engineering, Georgia Institute of Technology, Atlanta, Georgia, United States of America; 3 Wallace H. Coulter Department of Biomedical Engineering, Georgia Institute of Technology, Atlanta, Georgia, United States of America; 4 George W. Woodruff School of Mechanical Engineering, Georgia Institute of Technology, Atlanta, Georgia, United States of America; 5 Department of Infectious Diseases, College of Veterinary Medicine, University of Georgia, Athens, Georgia, United States of America; Uniformed Services University of the Health Sciences, United States of America

## Abstract

Lymphatic Filariasis, a Neglected Tropical Disease, is caused by thread-like parasitic worms, including *B. malayi*, which migrate to the human lymphatic system following transmission. The parasites reside in collecting lymphatic vessels and lymph nodes for years, often resulting in lymphedema, elephantiasis or hydrocele. The mechanisms driving worm migration and retention within the lymphatics are currently unknown. We have developed an integrated *in vitro* imaging platform capable of quantifying *B. malayi* migration and behavior in a multicellular microenvironment relevant to the initial site of worm injection by incorporating the worm in a Polydimethylsiloxane (PDMS) microchannel in the presence of human dermal lymphatic endothelial cells (LECs) and human dermal fibroblasts (HDFs). The platform utilizes a motorized controllable microscope with CO_2_ and temperature regulation to allow for worm tracking experiments with high resolution over large length and time scales. Using post-acquisition algorithms, we quantified four parameters: 1) speed, 2) thrashing intensity, 3) percentage of time spent in a given cell region and 4) persistence ratio. We demonstrated the utility of our system by quantifying these parameters for L3 *B. malayi* in the presence of LECs and HDFs. Speed and thrashing increased in the presence of both cell types and were altered within minutes upon exposure to the anthelmintic drug, tetramisole. The worms displayed no targeted migration towards either cell type for the time course of this study (3 hours). When cells were not present in the chamber, worm thrashing correlated directly with worm speed. However, this correlation was lost in the presence of cells. The described platform provides the ability to further study *B. malayi* migration and behavior.

## Author Summary

Lymphatic Filariasis is the largest world-wide source of secondary lymphedema and is caused by parasitic nematodes that migrate to and dwell in the lymphatic system. The World Health Organization estimates that over 120 million people in 73 countries are currently infected, and a further 1.4 billion live in infection-prone areas. Infection is initiated when a mosquito deposits infective larvae on the skin of the human host. The larvae then penetrate the skin at the site of the bite and migrate to afferent lymphatic vessels feeding into lymph nodes, where they mature into adult worms. While a large portion of infected individuals remain asymptomatic, many individuals show signs of potent immune responses that result in diseases such as lymphedema, elephantiasis and hydrocele. Through mass drug administration it is possible to kill the microfilariae stage of the parasite, which is the infective form for the mosquito intermediate host. There are currently no proven treatments for the adult worm, thus making it difficult to treat the estimated 120 million people already infected. Understanding how and why these parasites migrate to and reside in the lymphatic system will further aid researchers in designing treatment strategies that interrupt this mechanism. Here we describe an *in vitro* platform that allows researchers to quantify the migration behavior and the effects of drugs while maintaining the worm in the presence of cells typical of the host infection site.

## Introduction

Lymphatic Filariasis (LF) is the single largest world-wide source of secondary lymphedema [1] and is caused by adult parasitic nematodes that target and dwell in the lymphatic system. An estimated 120 million people in 73 countries are currently infected, and a further 1.4 billion live in areas where filariasis is endemic [2]. Of the 120 million people harboring the parasites, 90% have *Wuchereria bancrofti*, while *Brugia malayi* and *Brugia timori* infections account for the other 10% [3]. All three parasites use mosquitoes as transmission vectors [4]. Infection is initiated when the host-seeking mosquito deposits an infective third-stage larva (L3) on the skin of the host during the process of obtaining a blood meal. The infective larvae then penetrate the skin at the site of the bite, presumably guided by chemoattractants [5], and migrate to the lymphatic vessels and lymph nodes of the host where after 6–12 months they mature into adult worms. The adult worms may reside within the lymphatic system for years before the host shows any clinical manifestations such as lymphedema, hydrocele, elephantiasis, chyluria and compromised immunity [6]–[12]. Following mating in the lymphatics, the parasites release live progeny called microfilariae, which circulate in the bloodstream. These microfilariae can then be ingested by a mosquito during a blood meal, where they undergo development to form L2 and finally L3 larvae. Hence, the life cycle continues [7].

In the year 2000, the World Health Organization (WHO) launched the Global Alliance to Eliminate Lymphatic Filariasis (GAELF). The GAELF has been one of the most rapidly expanding global health programs in the history of public health with the goal of eliminating LF by 2020 through annual mass drug administration (MDA) [2], [4], [13], [14]. While killing the adult worms is considered one of the best strategies, the drugs used in MDA are only effective at killing microfilaria, and not the adult worms [15]–[20]. Thus, breaking the cycle of transmission has proven to be difficult. Additionally, these treatment strategies provide no relief for the estimated 120 million people already infected. As we move from controlling the disease to eliminating it, an understanding of the mechanisms by which L3 filarial parasites target and migrate towards lymphatics and how they behave in the presence of the lymphatic environment will be crucial in developing treatment strategies targeting the migration process as well as the lymphatic-inhabiting adult worms.

*In vitro* experiments suggest *B. malayi* induce local lymphatic remodeling via up-regulation of matrix metalloproteases (MMPs) [21] and actively secrete proteins to modulate immune function and evade detection [10]. Experiments with L3 *B. pahangi*, nonhuman filarial parasites, suggest sera isolated from mammals preferentially spur chemotaxis, possibly guiding worm penetration into the host at the bite site [5]. Additionally, experiments with intradermally injected *B. pahangi* exhibit differential gene expression compared to intraperitoneal injection [22]. These experiments suggest filarial parasites actively sense and respond to the local cellular microenvironment. Nematodes respond to a variety of different stimuli. Chemotaxis mediated by movement toward or away from chemical gradients, plays an important role in food- and mate-finding, and other aspects of nematode interactions. In very few cases have attractive substances been isolated and identified [23]. There is currently no high throughput *in vitro* imaging platform that allows researchers to quantify the complex interactions between these parasites and their multicellular host environment. Understanding how filarial worms interact with the multicellular microenvironment may reveal how they target and migrate towards the lymphatic system, and why they reside in it. This will provide invaluable insight for the anti-parasitic drug community and aid in the development of drugs that target the migration process and adult worms which will greatly aid in MDA elimination efforts. Additionally, it could lead to insight as to how worms utilize the unique environment of the lymphatic to enhance drug resistance and immune evasion.

Assays have been developed in recent years that quantify worm migration, development, behavior and viability [24]–[37]. Existing worm trackers either use the centroid position [33], [38]–[40] of the worm or a “skeleton” of the worm's shape [26], [27], [30], [41]–[43] to track its location. Centroid-based trackers define worm position as the geometric center of the segmented worm in each video frame. They can follow multiple worms at low magnification or, with the aid of a motorized x-y stage and feedback control, they can follow single worms over multiple hours [38], [39]. The throughput of such trackers can be increased by operating several setups in parallel [44]. Centroid-based trackers provide limited information about the details of worm behavior such as thrashing. Skeleton-based trackers, by contrast, generally operate at high magnification and derive a skeleton of each worm from segmented images. These skeletons provide extensive information about behavior. Existing assays rely on motorized x-y motorized stages, read only single wells at a time, are low-throughput, and do not offer quantitative regional based tracking. While many of these systems have extensive uses, there is no current integrated platform that is capable of quantifying migration and regional cell-proximity based behavior of multiple worms in a multicellular microenvironment at high magnification.

Here, we describe a scalable platform that can track multiple worms in parallel, and extract key parameters describing migration and regional based behavior using a novel co-culture system which exposes a single L3 *B. malayi* worm to both lymphatic and dermal layer cell types. The application can process multiple worms simultaneously without user intervention, allowing for long-term experiments in a CO_2_ and temperature controlled environment. This system can be used to assay large parasites such as filarial parasites and study their targeted migration towards a variety of desired cell types. Our system is scalable for a variety of multi-well devices providing the ability to alter the worm environment for high-throughput drug screening. In its current 7-lane configuration, we characterized the behavior and tracked the migration patterns of L3 *B. malayi* in the presence of cell types specific to the human interstitium by quantifying four key parameters; 1) speed, 2) thrashing, 3) percentage of time spent in a cell region, and 4) persistence ratio. Furthermore, we validated the platform's sensitivity to worm behavior by quantifying the effect of the common anthelmintic drug levamisole (in the form of tetramisole) [45], [46] on L3 *B. malayi*.

## Methods

### Brugia malayi Culture

Freshly isolated L3 *B. malayi* parasites were obtained from the National Institutes of Health Filarial Research Reagent Resource (FR3) [47] at the University of Georgia (Athens, GA). Worms were rinsed in 5 successive washings with Endothelial Basal Medium (EBM) (Lonza, New York) supplemented with 20% FBS (Atlanta Biologicals Lawrenceville, GA), 1% Glutamax, 1% Penicillin-Streptomycin-Amphotericin (Gibco, New York), 25 mg/mL cyclic-AMP and 1 mg/mL hydrocortisone acetate (both from Sigma, St. Louis, MO). The worms were then maintained in 10 mL of EBM at 37°C in a 5% CO_2_ incubator for at least 18 hours prior to experimentation.

### Cell Culture

Lymphatic endothelial cells (LECs) were obtained through isolation from human neonatal foreskins via immunomagnetic separation using the LEC marker podoplanin as described previously [48]. The LECs were expanded in T75 flasks that had been previously coated for 1 h with a collagen solution containing type I rat tail collagen (BD Biosciences, San Jose, CA) at a concentration of 50 µg/mL in 0.1% acetic acid (Sigma). The cells were grown in EBM (Lonza, New York) supplemented with 20% FBS (Atlanta Biologicals), 1% Glutamax, 1% Pencillin-Streptomycin-Amphotericin (Gibco), 25 mg/mL cyclic-AMP, and 1 mg/mL hydrocortisone acetate (both from Sigma). LECs were split at 80–90% confluence and were used in experiments either at passage 8 or 9. Human dermal fibroblasts (HDFs) were cultured in Dulbecco's Modified Eagle Medium (DMEM) (Lonza) supplemented with 10% FBS and 1% Pencillin-Streptomyacin-Amphotericin. HDFs were split at 80–90% confluence and were used in experiments at passage 14.

### Decision Chamber

**Figure 1:**
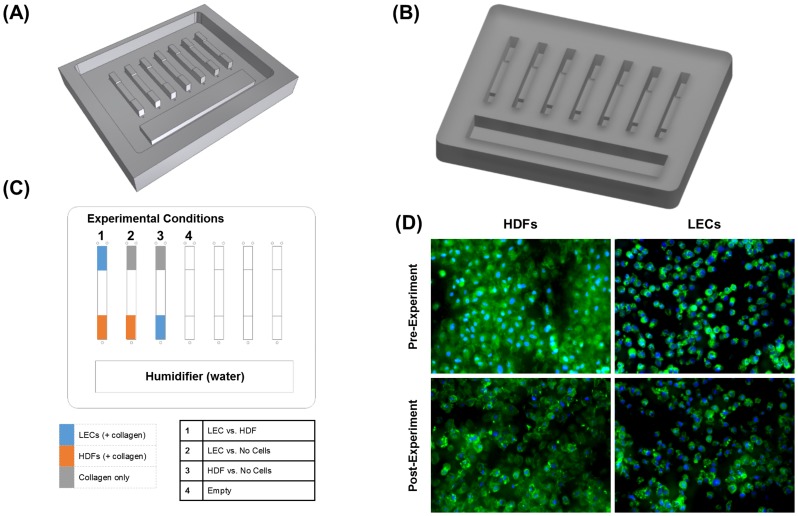
A worm-cell coculture device for studying nematode migration and behavior in a multicellular microenvironment. (**A**) A rendering of the aluminum mold used to cast the PDMS device (**B**) A rendering of the PDMS-based coculture device consisting of seven parallel lanes. (**C**) A top-view schematic showing the various regions discussed in this study. Each cell region occupies approximately 25% of the total lane area. The ‘humidifier’ is filled with water in order to limit media evaporation, flow lanes are included in the device for future flow-related experiments but were not utilized in the current study. (**D**) LECs and HDFs cultured in the device before and after a 3-hour exposure time to the worm (blue  =  nucleus, green  =  Actin). Spreading of the cells was less pronounced than what would be seen on polystyrene plates due to the surface roughness of the machine PDMS mold. The presence of the worms in the device did not seem to affect cell viability.

The mold for the decision chamber was designed in Autodesk Inventor 2013 and milled in 6061 aluminum (Figure 1A). To construct a device, poly(dimethylsiloxane) (PDMS) with a 10∶1 ratio of base to curing agent (Sylgard 184, Dow Corning) was poured in the mold, degassed for 20 minutes using a vacuum chamber, and then cured at 60°C for a minimum of 8 hours. The mold featured seven equidistant linear lanes, which allowed for the culture of two different cell types in each lane. Each cell region (referred to as ‘well’ throughout) started with a 200 µm down step which allowed for additional fluid retention in the region during the cell seeding process but did not significantly impede worm movement. In addition, only the ‘well’ region was adherent to cells due to a collagen coating. Regions outside of the well (PDMS surface) did not allow cell adhesion and thus prohibited cell migration. The device dimensions were chosen to provide the worm, which is placed in the center, with equal access to the cell types being evaluated in tandem (Figure 1B and C). The dimensions of each lane were 30×3 mm. Each cell region occupied 22.5 mm^2^ (25% of the total lane area). Full schematics and CAD files of the mold are available upon request.

### User Interface

A graphical user interface (GUI) was created in LabVIEW 2013 (National Instruments, Austin, TX). The GUI allows the user to select the number of lanes to track, the duration to track each worm for during an imaging cycle and the total experiment time. After setting the initial parameters, user interaction was no longer required. The developed LabVIEW virtual instrument was also used to interface the microscope control dynamic link library (dll) with the rest of the imaging program.

### Microscope Control

The Zeiss MTB2004 64 bit SDK along with Visual Studio 2010 (Microsoft, Redmond, WA) were used to create a C# dynamic link library (dll) allowing full control of a Zeiss AxioObserver Z1 inverted microscope (Carl Zeiss, Jena, Germany) along with a motorized x-y stage. The dll was accessed using a LabVIEW virtual instrument. The developed library allowed for full control of all microscope features including; filter-wheel, objectives, light intensity, incubation temperature and CO_2_ levels, and x-y-z position.

### Image Acquisition

Video frames required for centroid location and post-acquisition analysis were captured with a Guppy Pro CCD camera (Allied Vision Technologies, Newburyport, MA) at 15 frames/second (fps) with a resolution of 640×480 pixels. The program ran on a Lenovo Intel dual-core CPU with 4 Gb of RAM (Lenovo, Morrisville, NC) running Windows 7 64 bit. For the set of experiments carried out in this study a 2.5× microscope objective was used with a 0.5× C-mount camera adapter giving a total effective magnification of 12.5× (accounting for the 10× microscope phototube).

### Tracking Algorithm

**Figure 2:**
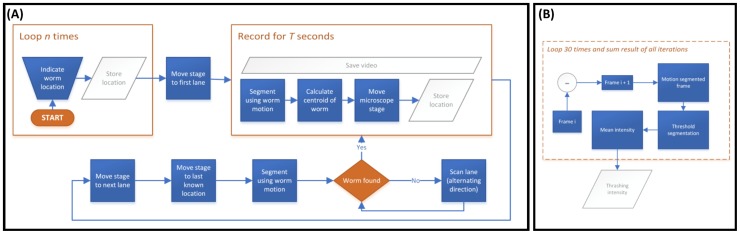
Block diagram of the worm tracking and thrashing algorithms. (**A**) The procedural steps involved in the worm tracking algorithm. Initial worm location in each lane is determined manually. The program then starts the process of acquiring video and cycles through all the lanes by moving to the last known worm location. If the worm is not found, then a linear scanning process initiates in order to find the worm. The scanning process alternates the start position, hence the direction of movement, in order to negate the effect of the microscope stage movement on worm displacement. (**B**) The thrashing algorithm takes two consecutive images and subtracts them to remove both background and all static features. The resulting image represents degree of worm movement during the time period separating the two frames (∼66 ms for a frame rate of 15 fps). The segment is then thresholded and the mean intensity of the resulting image calculated. The mean intensity is summed for the entire length of an imaging cycle (2 seconds) and the resulting values are normalized to obtain the ‘*thrashing index*’ metric.

Video was acquired at 15 fps. Frames were stored as individual 8-bit compressed TIFFs. Each two consecutive frames were subtracted to obtain a difference image representing motion-based segmentation of the worm. The resulting image was then binarized using a clustering based thresholding approach using the included blocksets in the Vision Development Module 2013 (National Instruments). Small particles were then filtered out and a binary image convex hull function was applied. The centroid location of the resulting segment was then calculated and taken as being a close approximation to the worm centroid. The motorized stage was then moved in the x-y plane to align the calculated centroid with the static center of the camera field of view (FOV) thus moving the worm to the center of the FOV. This process is repeated every two seconds, while video of the worm is acquired, and the x-y position of the stage (representing the worm location) is stored along with the corresponding time-stamp. The program then moves the stage to the next lane in which the center is pre-determined and repeats until all the lanes have been covered. When the program returns to a previously imaged lane, it moves to the last known location of the worm. If the worm was not found, it begins a scanning process from either the top or bottom of the lane until the worm is found. The scanning process alternates the direction of scanning to negate the effect of stage movement on worm displacement. Video is only stored when the worm is in the FOV. Figure 2A provides a block diagram representing pseudo-code for the worm tracking implementation. Full code is available upon request.

### Experimental Procedure

The PDMS decision chamber was rinsed with 70% ethanol followed by deionized water and left in the oven at 60°C to dry for 30 minutes. The chamber was then UV treated in a UV cleaner for 30 minutes to increase surface hydrophilicity. The cell regions of the lanes were treated with 50 µg/mL of Type I Rat Collagen (BD biosciences) in sterile 0.1% acetic acid for 1 hour at room temperature. Either human dermal fibroblasts or lymphatic endothelial cells were seeded in the well at a density of 20,000 cells/well in 100 µL of EBM, and allowed to adhere for 30 minutes at 37°C. Regional selection for cell seeding was randomized to remove any bias inherent to the chambers that might preferentially direct worm taxis. The chamber was then centrally flooded with EBM and cultured for 2 hours at 37°C. Thirty minutes prior to an experiment, the EBM was replaced with fresh EBM. Single worms were then introduced into the center regions via pipette. Worms were individually centered in the field of view and location was recorded within the user interface software. After all worms had been centered and located, the tracking system was initiated and worms were tracked for 3 hours. A halogen light source was used to illuminate the worm being imaged. The worm was only exposed to the light source when being tracked and was in complete darkness during all other time of the experiment (hence better mimicking *in vivo* light conditions). We used the device with four experimental conditions using two cell types as shown in Figure 1C. Tetramisole experiments followed the same procedure, except tetramisole (Sigma) was added to a lane to yield concentrations of 1.2 mM or 2.4 mM.

### Post-acquisition Analysis

Tracking the centroid and recording video for each worm within the device allowed us to extract various metrics describing worm motility both in the context of the entire lane as well as specific regions. ‘*Speed*’ was calculated for each section of the device, to compare worm speed in different environments, and over the entire tracking period. Thrashing measurements were carried out by subtracting two subsequent frames with an interval of 66 ms apart to obtain a difference image. The resulting image was then binarized using a metric based thresholding approach. The mean intensity of the entire image was then calculated and summed for a complete cycle (30 frames total) to obtain the ‘*thrashing index*’ metric (Figure 2B). In addition to the two motility metrics, the ‘*percentage of time spent*’ in any given region was calculated. The ‘*persistence ratio*’ was calculated by subtracting the final location of the worm at the end of the experiment from the location at the start and dividing by the total displacement of the worm during the entire 3-hour experiment. To determine the extent that the persistence ratio might change over time, the persistence ratio was also calculated over a 10 minute non-overlapping sliding window. Algorithms for determining the speed, time spent and persistence ratio were written in MATLAB 2013 while LabVIEW 2013 was using for the trashing metric.

### Statistical Analysis

A Kruskal-Wallis nonparametric test followed by Dunn's test to correct for multiple comparisons was used for statistical analysis of the *percentage of time spent* in each cell region. All other statistical tests were performed with a one-way ANOVA followed by a Tukey test to correct for multiple comparisons. All statistical analyses were performed in GraphPad Prism 6. P≤0.05 was considered statistically significant. Graphical P value designation was as follows: (P≤0.05)  =  *, (P ↕ 0.01)  =  **, (P≤0.001)  =  *** and (P≤0.0001)  =  ****. All data is presented as mean ± standard deviation. Sample number is indicated in each figure caption where applicable.

## Results

### A Scalable PDMS-Based Coculture Choice Chamber

The choice chamber allowed for co-culture of two cell types along with the L3 *B. malayi* thus creating a multicellular microenvironment for the worm. The aluminum mold used for the casts allows for repeated manufacturing of devices for a large number of experiments (Figure 1A). Microgrooves resulting from machining the mold had the advantage of providing a relatively rough surface, thus increasing friction, to potentially facilitate worm movement. Made of PDMS, the chamber was both biocompatible and optically clear allowing for both transmission and reflective imaging using an inverted microscope. The linear parallel lane configuration is also scalable to include more lanes per device if needed. The humidifying chamber, filled with sterile water or PBS, limited evaporation of the media (Figure 1B). Cells remained intact at the conclusion of the experiment with minimal signs of cytoskeletal remodeling as can be seen by the green actin stain of a representative image of the LECs and HDFs (Figure 1D).

### An Automated Imaging Platform for Quantifying Speed, Thrashing and Migratory Behavior

**Figure 3:**
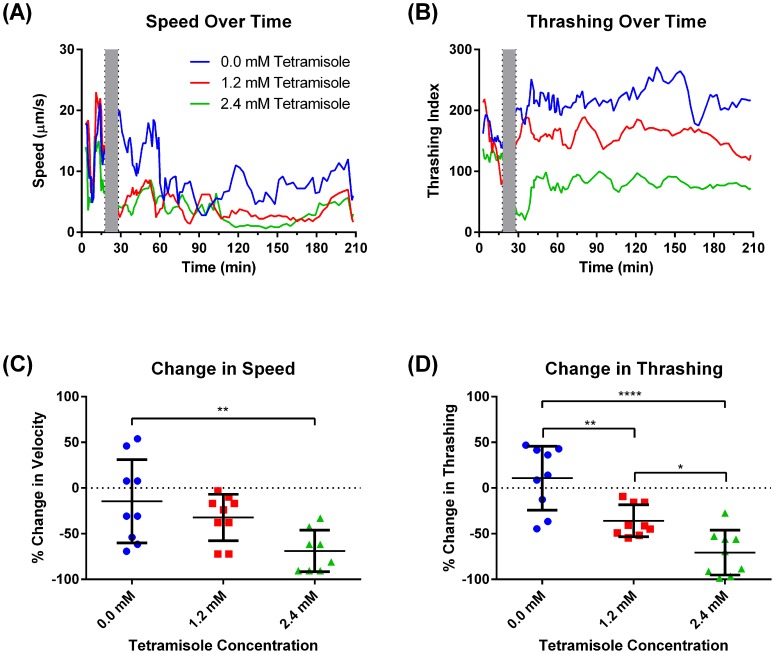
Tetramisole reduces both worm speed and thrashing. Representative speed (**A**) and thrashing (**B**) over a 3-hour experimental period for three worms under different concentrations of tetramisole (0.0, 1.2 and 2.4 mM) as measured with our platform. Tetramisole is a known paralytic agent that affects nematode thrashing. The gray interval represents a 10-minute gap when the drug was added and imaging session restarted. Our platform can detect changes in both worm speed (**C**) and thrashing (**D**) after drug administration. While both speed and thrashing decreased as tetramisole concentration was increased, the thrashing metric was more sensitive to the changes in tetramisole concentration. N = 9, error bars represent standard deviation. Sample videos provided in supplemental materials (S1, S2 and S3).

We developed an *in vitro* imaging platform that was used to study the migration behavior of nematodes in a multicellular microenvironment. The tracking algorithm provided the capability of imaging multiple worms under high magnification by imaging one worm at a time and then moving on to the next. If the worm was lost, then a search process was initiated to find the worm. A two-second video sequence was recorded along with the location of the worm during each cycle (Figure 2A). The system was built around a fully controllable environment in terms of both atmospheric CO_2_ levels and temperature, which made it ideal for long-term experiments requiring prolonged monitoring and quantification. With our current 7-lane configuration and 2-second imaging window for each worm it took approximately 120 seconds for a full cycle (in which 7 worms were tracked and imaged) with the main time spent on the search algorithm to find the worm if it had left the FOV of the last known location. To demonstrate the sensitivity of the two metrics for detecting changes in worm behavior, we exposed L3 *B. malayi* to tetramisole, a known anthelminthic, and showed that both speed and thrashing intensity decreased as a function of tetramisole concentration (Figure 3). At a concentration of 1.2 mM there was a 33% reduction in worm speed and a 37% reduction in thrashing. These values were increased to 70% for speed and 72% for thrashing when the concentration was increased to 2.4 mM. This change in speed was observed within 10 minutes of treatment with the tetramisole.

### L3 *B. malayi* Motility Is Altered in the Presence of Cells

**Figure 4:**
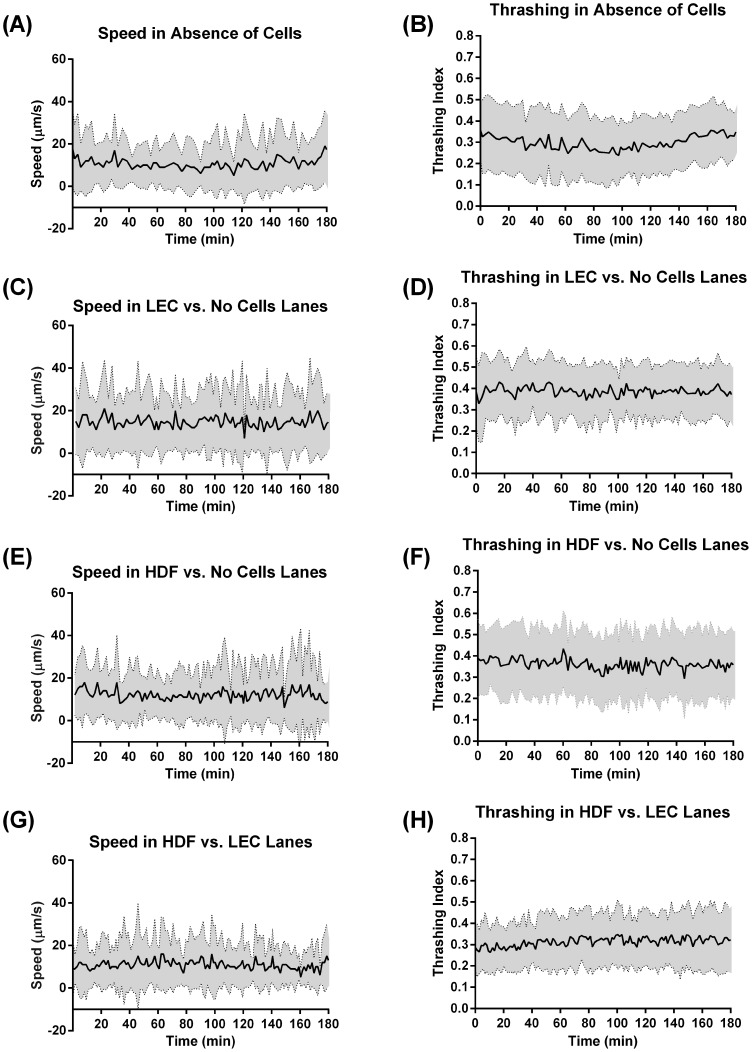
L3 *B. malayi* speed and thrashing, over a 3-hour experimental session, remain constant. (**A–H**) Speed and thrashing plots over a 3-hour period under each lane condition. 1) *Absence of cells*: no cells (nor collagen coating) present in the lane, 2) *LECs vs. no cells*: LECs on one well and collagen coating on the other, 3) *HDFs vs. no cells*: HDFs on one well and collagen coating on the other, and 4) *HDFs vs. LECs*: HDFs on one well and LECs on another. A relatively flat trend was seen for all cases indicating worms were viable and showed consistent behavior throughout the experimental time-frame. N≥28, error band represents standard deviation.

**Figure 5:**
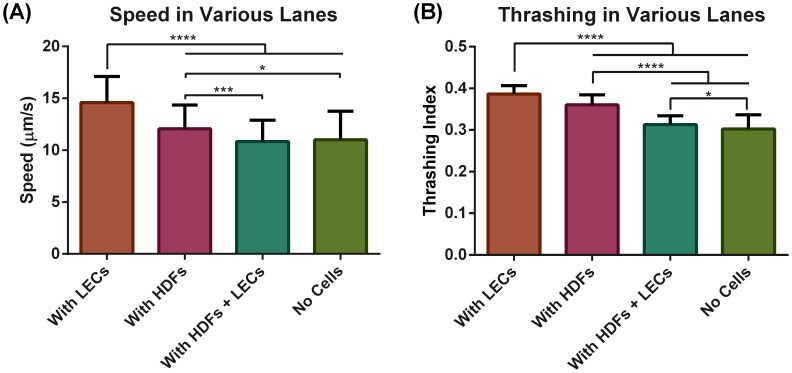
L3 *B. malayi* exhibit increased speed and thrashing in the presence of cells. Average speed (**A**) and trashing (**B**) of the worms under different conditions: When there are 1) *only LECs* in the lane 2) *only HDFs* in the lane 3) *both HDFs and LECs* in the lane, and 4) *no cells in the lane*. The worms were most active when in the LEC lane. They were also more active when only one of the cell types was present compared to both being present in the same lane. N≥28, error bars represent standard deviation.

**Figure 6:**
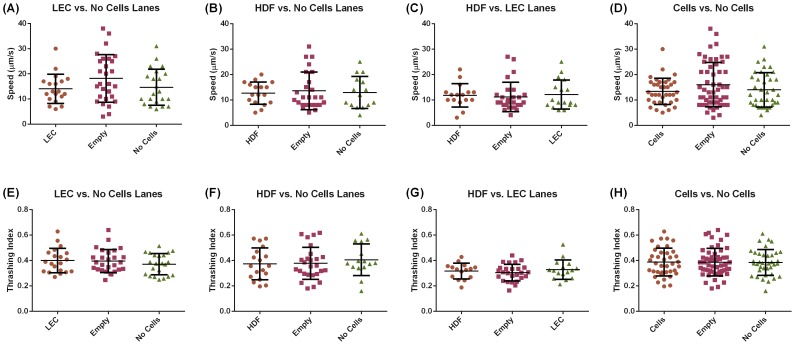
L3 *B. malayi* speed and thrashing are independent of physical contact with cells. Speed of worms and thrashing behavior when in physical contact with LECs (**A, E**), HDFs (**B, F**), HDFs + LECs (**C, G**) and combined data (**D, H**). No statistical differences were observed. N≥28, error bars represent standard deviation.

**Figure 7:**
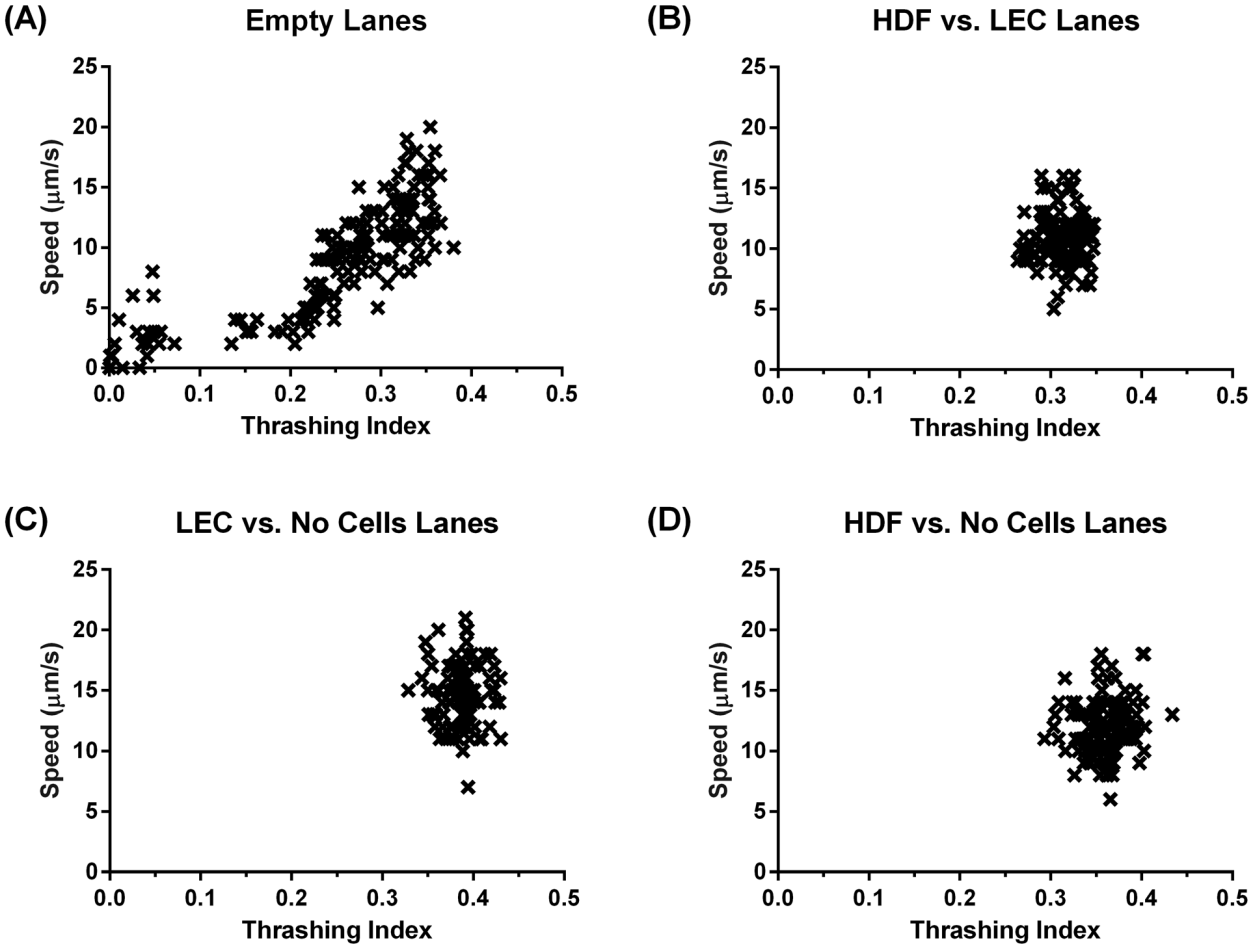
L3 *B. malayi* speed and thrashing are correlated in an empty lane, but not when cells are present. (**A**) In the empty lanes thrashing correlated with speed (Pearson correlation coefficient; r = 0.81). (**B–D**) There was no correlation when there were cells in the lane (r = 0.006, −0.049 and 0.12 respectively) which covered 25% of the total lane area. N≥28.

The location data along with the video sequences allowed us to extract both the speed and thrashing intensity for each worm over time demonstrating that the L3 *B. malayi* maintained relatively constant motility throughout the experiment with a speed of around 10–15 µm/s (Figure 4). Worm speed was highest in the presence of LECs followed by HDFs (15 µm/s and 12 µm/s respectively). No difference in speed was found when both cell types were present versus no cells at all (Figure 5A). Thrashing was highest in the presence of LECs followed by HDFs and then when the two cell types were both present (Figure 5B). While the overall presence of cells within the device enhanced worm motility, there was no difference in speed or thrashing when the worm was in physical contact with the cells, i.e. when the worm was in a given cell region (Figure 6). In addition, we found that in the case of a completely empty lane (no cells or collagen) the thrashing intensity correlated with speed to a high degree (Pearson correlation coefficient of 0.81) but the two metrics were no longer correlated when cells were present (Pearson correlation coefficients of 0.006 for HDFs + LECs, −0.049 for LECs alone and 0.12 for HDFs alone, Figure 7).

### L3 *B. malayi* Do Not Show Targeted Migration towards LECs or HDFs

**Figure 8:**
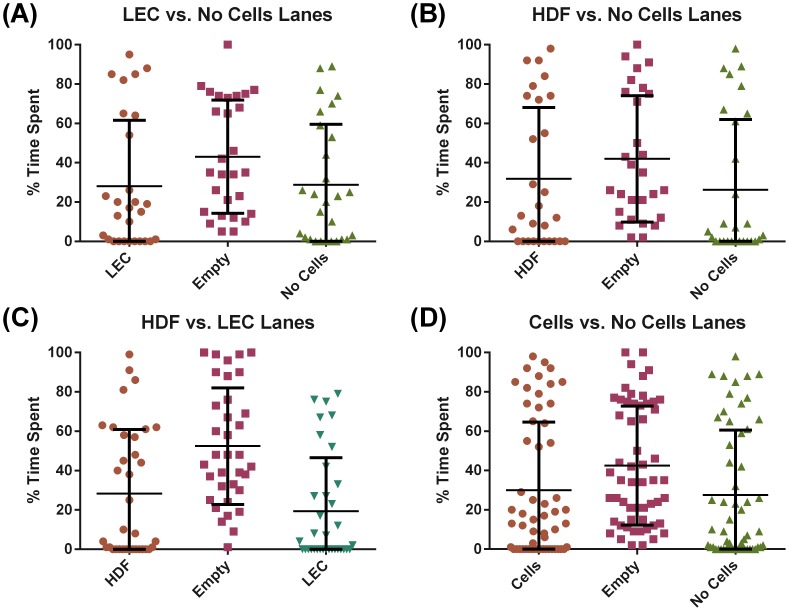
No difference in percentage of time spent by L3 *B. malayi* in each lane region. (**A–D**) Cell (HDF or LEC) and no cell (only collagen coating) areas each cover 25% of the lane, while the empty region is 50% of the area. No statistical differences were observed when accounting for area differences. N ≥ 28, error bars represent standard deviation.

**Figure 9:**
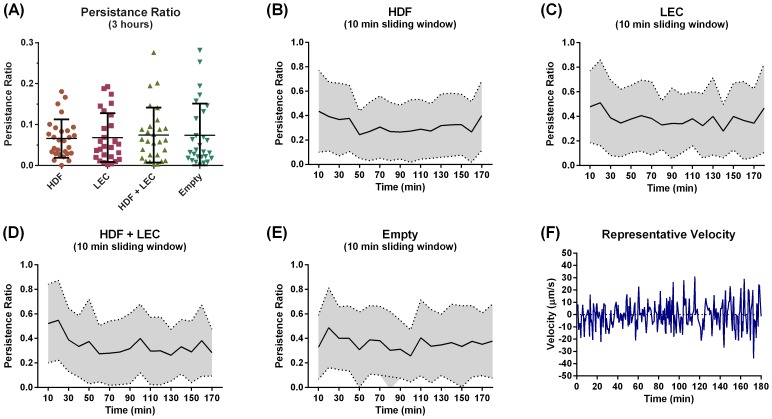
L3 *B. malayi* do not show targeted migration towards LECs or HDFs. (**A**) The persistence ratio calculated over the entire time of the experiment (3 hours). (**B–E**) The persistence ratio calculating for a 10 minute non-overlapping sliding window. (**F**) A representative velocity plot for one worm illustrating the randomness in directionality. N≥28, error bars and bands represent standard deviation.

In order to determine whether L3 *B. malayi* had a preference towards a certain cell type we quantified the percentage of time spent in each cellular region of the device. There was no preference towards a certain cell type as the worms spent equal time in all regions regardless of the culture conditions (Figure 8). To quantify the presence of any targeted migration we calculated the persistence ratio and found that the worms had very low persistence regardless of the culture conditions, suggesting that the worms' migration, while rather active, was fairly random (Figures 9A–E). This lack of targeted migration is further illustrated by a tracing of a typical worm's velocity, which oscillates back and forth as the worm continuously migrates up and down the lane (Figure 9F).

## Discussion

We demonstrated a platform for monitoring long-term nematode migration related behavior in a complex multicellular microenvironment that is potentially scalable for high through-put drug screening. The image acquisition system is flexible and surpasses most other published systems in acquisition capability [49] (See Table S1). The platform can be used with any nematode, including *C. elegans*, which are the most widely used model for studying nematode migration and behavior, since both tracking and analysis are independent of worm size and shape. Video is captured using a 640×480 pixel resolution camera but is capable of using any NI Vision compatible camera. Experiments were performed at a frame rate of 15 fps while the system is configurable to run at 60 fps without any reduction in resolution. The graphical user interface (GUI) is easy to use and requires minimal user intervention. The set-up is scalable to include any given number of lanes with the only limitation given by the minimum required dimensions of the lanes in order to encompass the given worm size and how much ‘blind time’ is acceptable between successive imaging cycles. From our experiments with the current device dimensions, the addition of each lane adds an average of 17 seconds of blind time as the algorithm has an additional lane to scan and image. While the software is only compatible with current Zeiss manufactured microscopes, due to the fact that we utilized the Zeiss microscope SDK, it does provide us with full control of every part of the microscope. Due to the modular design of the control VIs, we can easily add full control of the fluorescent filter wheel, objective, focus, illumination and dual-camera ports for experiments requiring more complex image acquisition workflows. The PDMS-based choice chamber provides a cheap and robust platform for nematode behavioral assays in which their interaction with various cellular environments would be of interest. Although the worms are capable of moving on the surface of the PDMS a three dimensional matrix environment would better recapitulate the migratory environment the worm must traverse to reach the lymphatic [50]–[52]. This setup would provide the benefit of creating a more defined concentration gradient of any potential chemo-attractants released by cells, however, it is uncertain whether L3 B. *malayi* have the capability of moving through such an environment.

We demonstrated that L3 *B. malayi* exhibited an increase in motility, as defined by speed and thrashing, when cells are cultured with the worm. The worms seemed to be most motile when LECs were present, followed by HDFs and then followed by the two cell types together. Co-culture with specific cell types has been previously shown to enhance worm survival. Falcone et al. showed that by using Jurkat and HDFs as feeder cells, L3 *B. malayi* survival was dramatically increased and allowed L3s to mature into L4s *in vitro* [53]. Thus, the increased motility seen with our system could be resulting from the production of an (or several) important micronutrient or metabolite by the mammalian cells that is enabling increased worm motility. Given the limited information available regarding *B. malayi* sensory receptors we cannot at this time provide any further details regarding what molecules could be responsible for the modified behavior. What is interesting however, is that we have demonstrated that L3 *B. malayi* are capable of ‘sensing’ their multicellular environment, within minutes after exposure to cells, which suggests a cellular cue could play a role in determining their migration patterns and preference to reside in lymphatics. In addition to this rapid response in motility to cells, we demonstrated that tetramisole, a paralytic agent commonly used to reduce nematode motility, reduced *B. malayi* motility within minutes of adding the drug to the worms. Thus the kinetics of action of tetramisole on *B. malayi* is comparable to *C. elegans,* which at similar drug concentrations usually show decreased motility within 15 minutes [54], [55]. Thrashing is a common metric to quantify the effect of a drug in parasite studies and we have demonstrated that that our system can detect immediate changes in both thrashing and speed under a given drug. Therefore, the system can thus be used as a rapid drug screen for *B. malayi*, while at the same time culturing the worms in a multicellular environment. Additionally, there has been renewed interest in developing new methods of in vitro culture for filariasis nematodes that can support the support the entire life cycle of the worm. Traditional approaches have required worms to be cultured for weeks at a time to determine the culture supplements that result in the lowest worm death. Given that both cells and drugs produced a measureable (yet subtle) difference in worm behavior that could be immediately quantified, this system provides an ideal platform for pre-screening dozens of different culture conditions for optimizing an *in vitro* parasitic host environment including the presence of cell derived chemokines such as CXCL12 which was previously shown to enhance the growth of L4 filariae [56].

For the purpose of this study, we chose three widely used metrics to quantify behavior: speed, thrashing, and persistence ratio. In addition to these metrics, we determined the percentage of time spent at each cell type as a way of assessing whether L3 *B. malayi* had a certain preference for being in physical proximity to a given cell type. Our results indicated that L3 *B. malayi* did not have a preference towards a given cell type nor did they modify their motility (defined by both speed and thrashing) when in physical contact with LECs or HDFs. Interestingly, when cells are not present an increase in worm thrashing directly translates into an increase in worm speed, as suggested by the two parameters' high degree of correlation. The fact that this correlation is lost when cells are present, suggests that not only are the worms increasing their speed, but also that thrashing (as we have determined it) no longer is the driving mechanism determining worm migration. At the very least, the analysis capabilities of our platform provide the ability to discriminate between very subtle changes in behavior that otherwise would not be apparent with traditional approaches. We then attempted to determine whether *B. malayi* exhibited directional guidance to lymphatic endothelial cells and found that they had no preference towards either cell type tested (LECs and HDFs). While chemotaxis through a gradient of CCL21 released by lymphatic endothelial cells has been shown to promote dendritic cell migration to lymphatic vessels [50], [57], [58], there are no known chemotactic molecule-receptor pairs identified for filarial parasites, much less ones that involve a lymphatic chemokine. Such chemotaxis of the larvae to serum, as shown previously, would support the hypothesis that chemotaxis can drive targeted migration [5], [59], [60]. However using our platform, chemotaxis does not seem to be the main contributor to migration towards lymphatics. This phenomenon could be due to the fact that stable chemokine gradients are not formed in our device due to the high diffusion coefficient of relevant chemokines in cell culture media. Other factors in the *in vivo* environment not captured in the current iteration of the device might play a large contribution to migration including the contents of lymph, the presence of immune cells, and interstitial flow (which is always directed towards the nearest draining lymphatic and has been implicated in lymphatic-targeting for other cell types [52], [61], [62]). In addition, worm movement within the dermis might be random until the worm encounters a point of entry into the lymphatics (i.e. collecting lymphatic vessels) which are large enough to encompass the worm and fragile enough to be penetrated given their thin walls.

The described platform provides a tool for parasitologists to explore mechanisms that drive L3 filarial worms to target lymphatic vessels, to screen for the efficacy of potential new drug compounds, and to engineer *in vitro* environments that provide a more viable host for long-term worm culture. While in its current form our study provides valuable insight by quantifying L3 *B. malayi* behavior both in the presence and absence of dermal specific cells, the *in vitro* platform needs to be further expanded to better capture key biophysical and biochemical aspects that are essential to the host environment including flow and concentration gradients. *In vivo* flow conditions can be replicated by flowing media through the channels with the appropriate wall shear stress values. A stable diffusion gradient will be somewhat challenging without the incorporation of a 3D matrix but one possibility would be depositing an immobilized 2D gradient on the surface of PDMS [63] to test a given chemokine in question. While MDA has proven successful to an extent, the main limiting factor, second to non-compliance [64], is that the drugs used do not kill adult worms. Hence, it is crucial that as we move from control to elimination that we find new strategies to disrupt the transmission cycle. This shift requires understanding L3 *B. malayi* migration and the effects of drugs in an environment that mimics *in vivo* conditions with the goal of creating an environment close enough to the human host to ultimately culture *Wuchereria bancrofti*, the primary filarial species that is responsible for 90% of infections.

## Supporting Information

Table S1 Table adapted from Husson et al. comparing current worm trackers.
(DOCX)

Video S1 An example two-second video segment used by the tracking and quantification algorithms.
(AVI)

Video S2 Reduced L3 *B. malayi* motility after exposure to 1.2 mM of tetramisole.
(AVI)

Video S3 L3 *B. malayi* motility after exposure to 2.4 mM of tetramisole.
(AVI)
