# Supplementary material for: An Integrated In Vitro Imaging Platform for Characterizing Filarial Parasite Behavior within a Multicellular Microenvironment
Source: PLoS Negl Trop Dis. 2014 Nov 20;8(11):e3305. doi: 10.1371/journal.pntd.0003305 (PMC4238983; doi:10.1371/journal.pntd.0003305)
Supplement: Table S1 — Table adapted from Husson et al. comparing current worm trackers. (DOCX) [file pntd.0003305.s001.docx]

| **Name** | **Worm Tracker 2.0 (Schafer lab)** | **Nemo (Tavernarakis lab)** | **The Parallel Worm Tracker (Goodman lab)** | **OptoTracker (Gottschalk lab)** | **Multimodal illumination and tracking system (Lu lab)** | **CoLBeRT (Samuel lab)** | **The Multi Worm Tracker (Kerr lab)** | **Opto-mechanical system for virtual environments (Lockery lab)** | **Filarial Imaging Platform (Dixon lab)** |
| --- | --- | --- | --- | --- | --- | --- | --- | --- | --- |
| **Single/Multi Worm** | Single | Single | <50 | <50 | Single | Single | <120 | Single | <50 |
| **Adaptable** | Yes, supports x-y stages by three different vendors, as well as other camera systems (i.e. USB cameras) | Yes, code open for changes, supports other camera systems (i.e. USB cameras) | Yes, code open for changes, supports other camera systems (i.e. USB cameras) | Yes, code open for changes, supports other camera systems (i.e. USB cameras) | Yes, code open for changes, supports any projector and LabVIEW Vision compatible camera systems (i.e. USB cameras) | Yes, code open for changes | Yes, code open for changes, supports LabVIEW Vision compatible camera systems | NA | Yes, code open for changes, supports LabVIEW Vision compatible camera systems |
| **Optogenetic application** | No | No | No | Yes | Yes – 3 wavelengths | Yes | Yes | Yes | Yes |
| **Illumination type** | NA | NA | NA | Whole field | patterned; intensity adjustable – each wavelength independently | patterned | Whole field | patterned, intensity adjustable | Whole field |
| **X-Y Stage control** | Yes | No | No | No | Yes | Yes | No | Yes | Yes |
| **Measured parameters** | Skeleton and outline | Skeleton and outline | Centroid | Centroid | Skeleton and outline | Skeleton and outline | Skeleton and outline | Bright spot | Centroid |
| **Camera resolution/support for other resolution (pixel)** | 1280 × 1024/Yes | 800 × 600/Yes | 640 × 480/No, downsized if greater | 640 × 480/No, downsized if greater | 320 × 240/Yes, but reduced fps at higher resolutions | 1280 × 1024 /NA | 2352 × 1728/No | 4 quandrants photomultipliertube | 780 x 580/Yes |
| **Camera frequency/other supported (frames per second)** | 30/Yes | 40/Yes | 15/Yes | 15/Yes | 25/Yes | 50/Yes | 31/No | NA-PMT | 60/Yes |
| **Video stored** | Yes | Yes | Yes | Yes | Yes | Yes | No | Yes | Yes |
| **GUI** | Yes | Yes | Yes | Yes | Yes | Yes | No | Yes | Yes |
| **Microscope required** | No | No | No | No | Yes | Yes | No | Yes | Yes |
| **Required Hardware^*^** | X-Y Stage, camera | Camera | Camera | Camera, light source with shutter, filters | X-Y Stage, camera, projector, filters | X-Y Stage, Laser, DMD Array, frame grabber, camera | Camera, frame grabber, background light | PMTand centering device | Zeiss Compatible X-Y Stage, Camera |
| **Required software** | Java, ffdshow, MATLAB or [MCR](http://www.wormbase.org/db/get?name=MCR;class=Cell) | MATLAB (R13) + Image Processing Toolbox | MATLAB (R13) + Image Acquisition and Image Processing Toolbox | MATLAB (R13) + Image Acquisition and Image Processing Toolbox | LabVIEW (+ Vision) | MindControl (custom, C), MATLAB R2010a | LabVIEW (+ Vision), C++ (custom), Java | NA | LabVIEW (+Vision) Runtime, Zeiss SDK |
| **Cost estimation excluding software, computer and microscope (US$)** | 3,500 | 350 | 350 | 1600 | 10,000 | 16,000 | 7,000 | Commercial version available (PhotoTrack, ASI) | $700 |
| * Some cameras require a frame grabber and PCI card to communicate with LabVIEW or MATLAB; USB- or fire-wire cameras should work w/o these. *Adapted from Husson SJ, Costa WS, Schmitt C, Gottschalk A (2013) Keeping track of worm trackers. WormBook: 1–17. Available: http://www.wormbook.org/chapters/www_tracking/tracking.html.* | | | | | | | | | |
